# A structural and functional study on the 2-C-methyl-d-erythritol-4-phosphate cytidyltransferase (IspD) from *Bacillus subtilis*

**DOI:** 10.1038/srep36379

**Published:** 2016-11-08

**Authors:** Yun Jin, Zhongchuan Liu, Yanjie Li, Weifeng Liu, Yong Tao, Ganggang Wang

**Affiliations:** 1 Key Laboratory of Environmental and Applied Microbiology, Chengdu Institute of Biology, Chinese Academy of Sciences, Chengdu, 610041, China; 2 Key Laboratory of Environmental Microbiology of Sichuan Province, Chengdu, 610041, China; 3 University of Chinese Academy of Sciences, Beijing, 100049, China; 4 Chinese Academy of Sciences Key Laboratory of Microbial Physiological and Metabolic Engineering, Institute of Microbiology, Chinese Academy of Sciences, Beijing 100101, People’s Republic of China

## Abstract

2-C-Methyl-D-erythritol-4-phosphate cytidyltransferase (IspD) is an essential enzyme in the mevalonate-independent pathway of isoprenoid biosynthesis. This enzyme catalyzes 2-C-Methyl-d-erythritol 4-phosphate (MEP) and cytosine triphosphate (CTP) to 4-diphosphocytidyl-2-C-methyl-d-erythritol (CDPME) and inorganic pyrophosphate (PPi). *Bacillus subtilis* was a kind of excellent isoprene producer. However, the studies on the key enzymes of MEP pathway in *B. subtilis* were still absent. In this work, the crystal structures of IspD and IspD complexed with CTP from *B.subtilis* were determined. For the first time, the intact P-loop was observed in the apo structure of IspD enzyme. Structural comparisons revealed that the concerted movements of the P-loop and loops close to the active site were essential in the reaction catalyzed by IspD. Meanwhile, kinetic analysis showed that the CTP hydrolytic activity of IspD from *B.subtilis* was over two times higher than that from *Escherichia coli*. These results will be useful for future target-based screening of potential inhibitors and the metabolic engineering for isoprenoid biosynthesis.

Isoprenoids are a diverse range of natural products which play an important role in living organisms^1^. In practice, a lot of isoprenoids have been applied in pharmaceuticals, neutraceuticals, cosmetics and food, it is well known that the artemisinin and its derivatives are widely used to treat malaria^2^,^3^; taxol (paclitaxel) is effective in cancer therapy^4^,^5^. Traditionally, the isoprenoids used are mainly produced by natural extraction or chemical synthesis. However, limited yield and high cost are far behind the increasing need. Today, it is becoming more promising to produce isoprenoids in microbe. Isoprenoid biosynthesis depends on the essential metabolic precursors, isopentenyl diphosphate (IPP) and dimethylallyl diphosphate (DMAPP). To date, two distinct metabolic pathways have been elucidated for the formation of IPP and DMAPP, one is the mevalonate (MVA) pathway found in most mammals, plants and fungi^6^,^7^; the other one is the 2-C-Methyl-d-erythritol 4-phosphate (MEP) pathway found in most plant chloroplasts, algae, eubacterias, apicomplexan parasites, cyanobacterias and diatoms^8^,^9^,^10^,^11^.

MEP pathway is made up of seven subsequent enzymatic steps. It initiates with the formation of 1-deoxy-d-xylulose 5-phosphate (DOXP) by condensation of pyruvate and D-glyceraldehyde 3-phosphate catalyzed by the DOXP synthase (DXS). DOXP is then converted into MEP by the DOXP reductoisomerase (DXR). The third enzyme is 2-C-Methyl-d-erythritol-4-phosphate cytidyltransferase (IspD), 4-diphosphocytidyl-2-C-methyl-d-erythritol (CDPME) is formed from MEP by reaction with CTP^12^. The fourth enzyme of the MEP pathway is CDPME kinase (IspE)^13^, which mediates the formation of 4-diphosphocytidyl-2-C-methyl-d-erythritol 2-phosphate (CDPME2P) in an ATP-dependent reaction. The fifth step is the formation of 2-C-methyl-d-erythritol 2,4-cyclodiphosphate (MEcDP) by MEcDP synthase (IspF)^14^. In the last two steps, the MEcDP is converted into 4-hydroxy-3-mehtyl-butenyl 1-diphosphate (HMBPP) by 1-hydroxy-2-methyl-2-(E)-butenyl-4-diphosphate synthase (IspG), then, the HMBPP is catalyzed by 4-hydroxy-3-methyl-2-(E)-butenyl-4-diphosphate reductase (IspH) to yield IPP and DMAPP^8^,^15^,^16^,^17^.

Since the enzymes of MEP pathway in bacteria are highly conserved but show no homology to mammalian proteins^16^, all enzymes of MEP pathway in pathogenic bacteria are potential therapeutic targets for the treatment of important infectious diseases. Inhibitors were designed for the development of new antibiotics and herbicide^18^,^19^,^20^,^21^. On the other hand, the MEP pathway underlines the biosynthesis of valuable isoprenoids by bacteria cell, the enhancement of the MEP pathway resulted in higher yield of various products, including isoprene, lycopene^22^,^23^, artemisinic acid^24^, taxadiene^25^, carotenoids^26^, *et al*. Extensive studies on the key enzymes of MEP pathway would provide valuable information for production of isoprenoids by synthetic biology and metabolic engineering.

The IspD protein converts MEP to CDPME in MEP pathway^10^ (Fig. 1). The studies on IspD of *E. coli* (*Ec*IspD) revealed the stereochemical principles underlying the catalysis mechanism. Otherwise, the IspD from *Mycobacterium tuberculosis* and *Arobidopsis thaliana* were extensively studied for the development of anti-tuberculosis (TB) drugs and herbicide, respectively^19^,^27^,^28^. *B. subtilis* is a model organism in biological research; moreover, it has been applied for the production of various bioactive molecules as a cell factory^29^. It was reported that *Bacillus* was the highest isoprene producers out of the microorganism screened^30^. Interestingly, Zhao *et al*.^31^ have found that the genes of *dxs* and *dxr* from *B. subtilis* heterologously expressed in *E. coli* functioned more efficiently on the enhancement of isoprene production than the native ones. Therefore, it is essential to study the enzymes of the MEP pathway in *B.subtilis* for isoprenoids production.

The IspD protein from *B.subtilis* (*Bs*IspD) is of 37% homology to that of *E. coli* (*Ec*IspD), currently, the structure of *Bs*IspD is not known yet. In this study, the crystal structures of *Bs*IspD and *Bs*IspD/CTP-Mg^2+^ were determined. For the first time, the P-loop, which played important roles in enzymatic reaction process, was determined intactly in the apo structure of *Bs*IspD. Moreover, with the intact information on the P-loop, the conformational change of P-loop was discussed during substrate binding, intermediate forming and product releasing. Otherwise, CTP hydrolysis activities of *Bs*IspD and *Ec*IspD were compared *in vitro*, and the catalytic efficiency of *Bs*IspD was higher than that of *Ec*IspD. These results would show light on the metabolic engineering for isoprenoid biosynthesis.

## Results

### Overall structure

Two crystal structures of *Bs*IspD have been determined in orthorhombic crystal form with space group *P*2_1_2_1_2_1_ and *P*2_1_2_1_2, named apo form I and II, respectively (Table 1). In the apo form I, two molecules (molecule A and B) in the asymmetric unit were related by twofold sysmetry and formed a dimer. The two subunits can be superposed with a root mean square deviation (r.m.s.d) of 0.15 Å for 232 equivalent C^α^ atoms. In the apo form II, only one molecule (molecule C) was retained in the asymmetric unit, the functional dimer was formed by the sysmetry operation. Two flexible loop regions (residues 13–14, 226–232) were not built in the final model, because of the poor quality of the electron density. The two apo structures were well conserved. The chain-to-chain superposition for 223 C^α^ positions revealed the r.m.s.d between subunits C-A and C-B were 0.41 Å and 0.46 Å, respectively, while the comparison on the dimer gave an r.m.s.d of 0.59 Å for 446 pairs of C^α^ atoms. Meanwhile, the structure of *Bs*IspD was similar to that of *Ec*IspD. Superposition of the subunits of *Bs*IspD and *Ec*IspD (PDB entry 1inj) resulted in a r.m.s.d values range from 1.0–1.2 Å for 200 C^α^ depending upon which chains were aligned.

**Figure 2:**
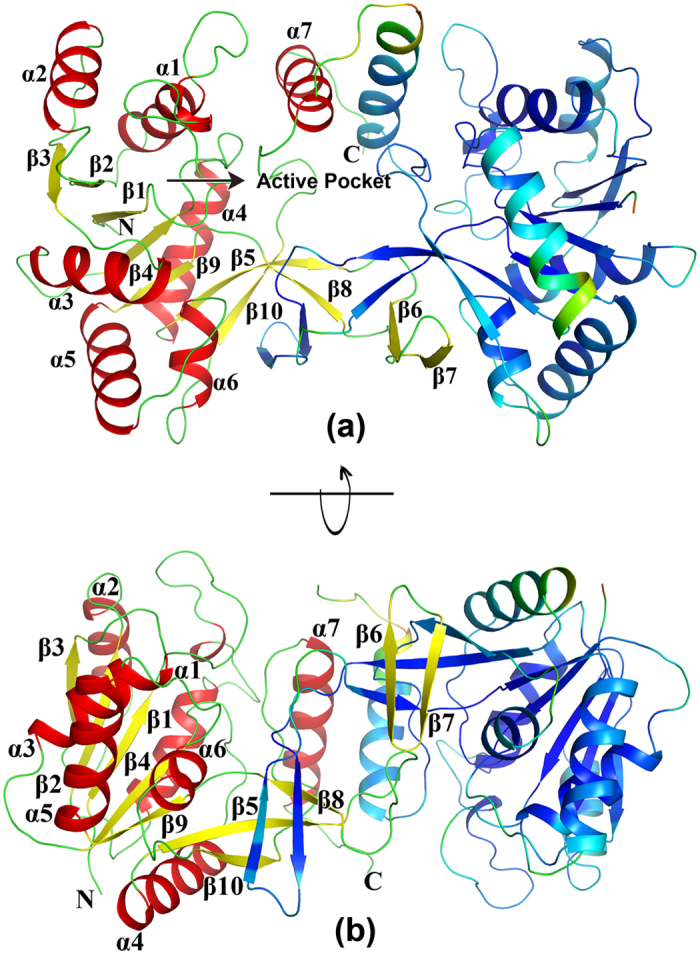
Cartoon presentation of the *Bs*IspD structure. The views have been chosen (**a**) to illustrate the position of the active sites in the homodimer without substrate and (**b**) to shown an overview of dimer formation. One chain has been drawn with rainbow coloring of B-factor, the other with the secondary-structure theme.

The subunit structure of *Bs*IspD was of a compact *α*/*β* fold from which a long *β*-meander extended. The core of the enzyme consisted of a seven *β*-sheets (*β*2, *β*1, *β*4, *β*9, *β*5, *β*8, *β*10) where all strands were parallel, apart from *β*8 and *β*2. The *β*-meander lay between antiparallel strands *β*6 and *β*7 and made the major contribution to the dimer interface, and the lesser contribution came from the side-chain interactions of the residues on the *α*-helix fragment at the C-terminus (Fig. 2).

### *Bs*IspD/CTP-Mg^2+^ structure

**Figure 3:**
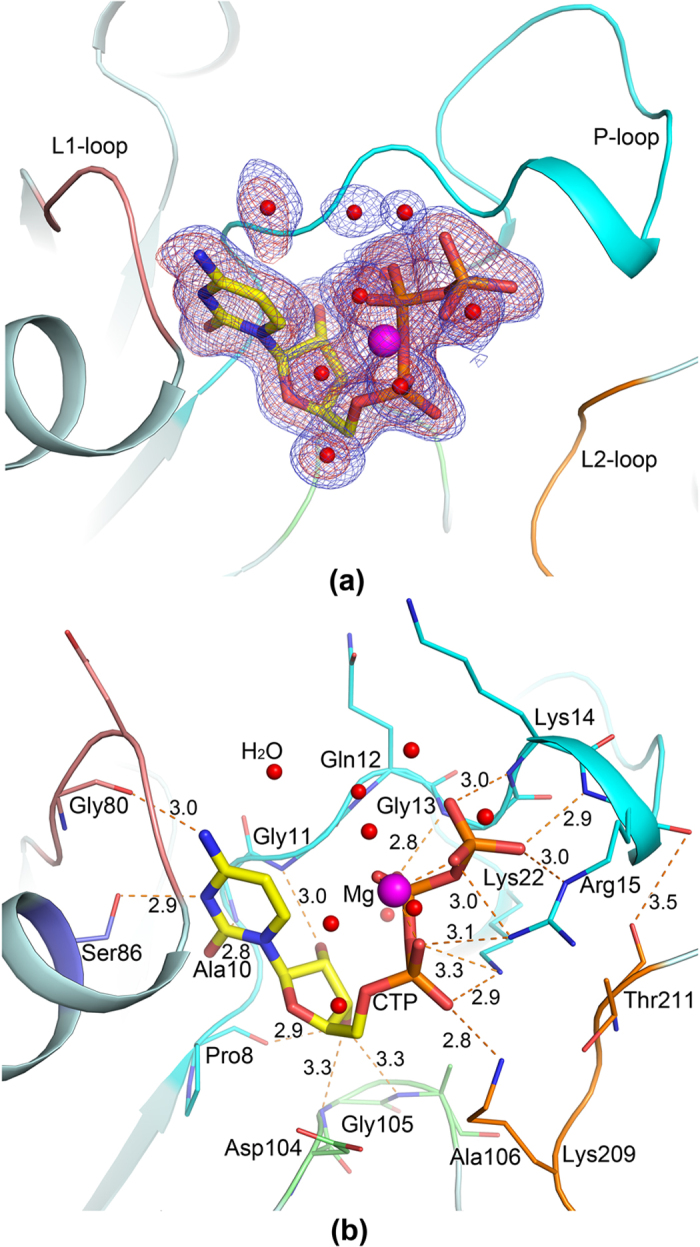
The interactions between CTP and *Bs*IspD. (**a**) Representative of the active pocket in the structure of *Bs*IspD/CTP-Mg^2+^ complex. The 2Fo-Fc electron density map (1.5σ level) around the CTP-Mg^2+^ was represented in blue, and the omit Fo-Fc map (3σ level) was represented in red. The residues 8–21, residues 79–83 and residues 208–212 were named as P-loop (cyan), L1-loop (magentas) and L2-loop (brown), respectively. (**b**) Representative of the interactions between CTP and *Bs*IspD. The CTP molecule was shown as stick model. The magnesium ion and the water molecules were shown as sphere. The relevant residues were shown as stick model. Hydrogen bonds were shown as orange dashes.

Furthermore, the structure of *Bs*IspD/CTP-Mg^2+^ complex was determined (Table 1 and Fig. 3a), the final model revealed two molecules in one asymmetric unit, named molecule D and E, respectively. These two subunits can be superimposed with a r.m.s.d of 0.63 Å for 215 C^α^. One CTP molecule and one magnesium ion were observed binding to the molecule D, whereas not in the molecule E. Structural analysis revealed the binding pocket in molecule E was exposed to the solvent much more than that in molecule D (Supplementary Fig. S1), which may affect the binding of CTP to molecule E. If no specific note is given, the discussion on *Bs*IspD/CTP interaction will be focused on molecule D. In the molecule D, the P-loop (residues 8–21) was fairly refined to a B-factor of 31 Å^2^ (Supplementary Fig. S2), whereas the P-loop region (residues 10–20) in the molecule E was disordered and not built in the final model. Moreover, the average B factor of the main-chain and side-chain of the molecule D was about 20 Å lower than that in the molecule E, which implied that molecule D was in more stable state than molecule E. Six residues (227–232) in the C-terminal of two molecules were missed in the final model.

In the crystal structure of *Bs*IspD/CTP-Mg^2+^ complex, as shown in Fig. 3b, the cytosine base (C) of CTP slotted between the flat peptide planes of P-loop and L1-loop(residues 79–82), three hydrogen bonding interactions were formed between cytosine base and residues Ala10, Gly80 and Ser86 (Ala10 N-C O2 = 2.8 Å, Gly80 O-C N4 = 3.0 Å, Ser86 OG-C N3 = 2.9 Å), all of these residues are strictly conserved in the IspD enzyme. In addition, the ribose was hydrogen bonded to Pro8, Gly11, Gly105 and Ala106 (Gly11 N-ribose O2′ = 3.0 Å; Pro8 O-ribose O3′ = 2.9 Å, Gly105 N-ribose O3′ = 3.3 Å and Ala106 N-ribose O3′ = 3.3 Å). The triphosphate wrapped around a magnesium ion, coordination bonds were formed between the magnesium ion and the oxygen atoms of *α*-, *β*-, *γ*-phosphate in CTP. Otherwise, hydrogen bonds/salt links were formed between the triphosphate and side-chain atoms of three strictly conserved basic residues (Lys22 NZ-PA O1A = 2.9 Å, Lys209 NZ-PA O1A = 2.8 Å; Arg15 NH2-PA O2A = 3.1 Å, Arg15 NH2-PG O3G = 3.0 Å, Arg15 NE-PG O1G = 3.0 Å), whereas the main-chain atoms of residues 13–15 directly bonded to the triphosphate as hydrogen-bond donors (Gly13 N-PB O2B = 2.8 Å, Lys14 N-PG O2G = 3.0 Å; Arg15 N-PG O1G = 2.9 Å).

In the apo form, the active pocket was in closed form with a narrow entrance, however, in the structure of *Bs*IspD/CTP-Mg^2+^ complex, the binding of CTP induced the conformational change of loops aroud the active pocket, this will be addressed in following sections.

### P-loop

**Figure 4:**
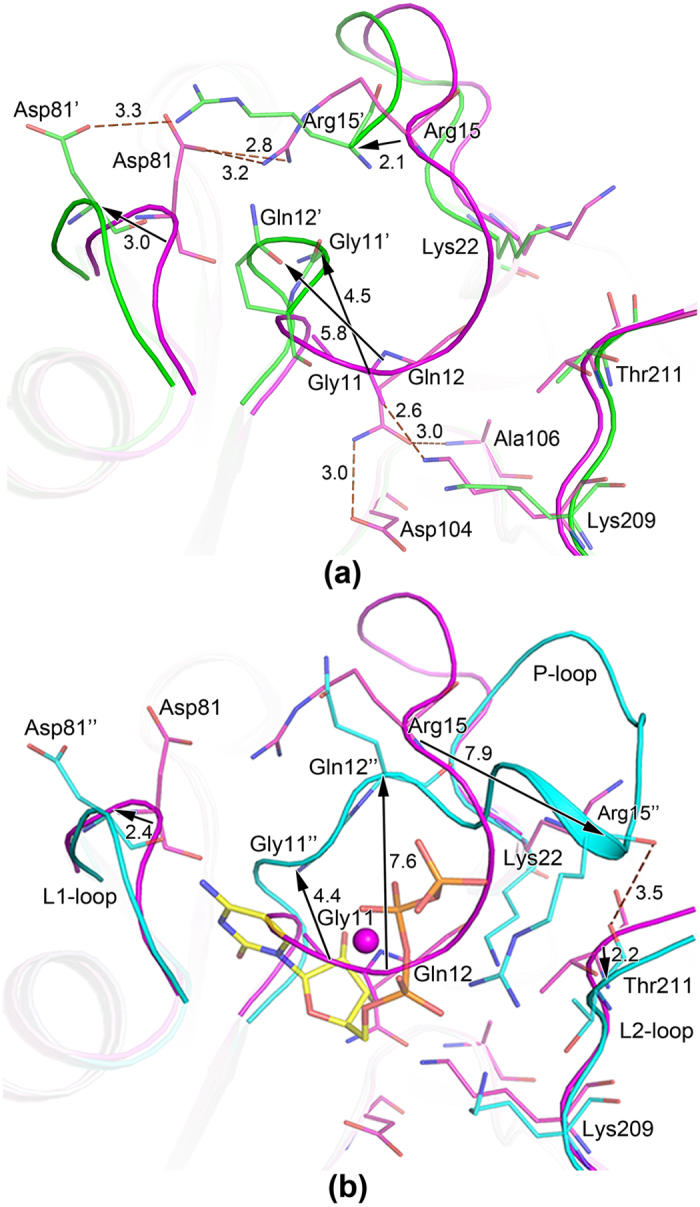
Conformational change of P-loop. (**a**) Superposition on two apo forms of *Bs*IspD. (**b**) Superposition of *Bs*IspD/CTP-Mg^2+^ complex and *Bs*IspD. Structrures of apo forms I, II and *Bs*IspD/CTP-Mg^2+^ complex were shown in cartoon and coloured by green, purple and cyan, respectively. The relevant residues were shown in stick model. The hydrogen bonds were shown as dashes, and moving distances of the Cα atoms were represented by black arrow.

The P-loop comprising residues 8–21 in the apo form I were refined satisfactorily, the B-factor of 30 Å^2^ was almost the same as the average B-factor of all the protein atoms (28 ^2^), this loop was highly conserved in the IspD enzymes (Supplementary Fig. S3).The residues Gly11 and Gln12 in the P-loop formed three hydrogen bonds with the adjacent residues Asp104, Lys209 and Ala106 (Gln12 NE2-Asp104 OD1 = 3.0 Å, Gln12 OE1-Ala106 N = 3.0 Å, Gly11 O-Lys209 NZ = 2.6 Å), all the three residues were part of the loops conformating the active pocket and strictly conserved in the IspD enzyme. Moreover, the residue Arg15 in the P-loop was hydrogen-bonded to the residue Asp81 in the L1-loop (Arg15 NH1-Asp81 OD1 = 3.3 Å, Arg15 NH2-Asp81 OD1 = 2.8 Å) (Fig. 4a).

In the structure of apo form II, although the residues 13 and 14 were not built, it was clear that the P-loop shifted upward from the active site, since the C^α^ atoms of Gly11, Gln12 and Arg15 shifted 4.5 Å, 5.8 Å and 2.1 Å, respectively, meanwhile, the side chain of Arg15 flipped to extend to Asp81 which moved about 3 Å. Therefore, the active site was more exposed to the solvent than that in the apo form I. Moreover, the L1-loop also rotated about 30° and moved about 3.0 Å. Here the residues of Gly11 and Gln12 were too far to interact with residues as described above, at the same time, only one hydrogen bond was formed bewteen Arg15 and Asp81 (Arg15 NH1-Asp81 OD1 = 3.3 Å) (Fig. 4a). The structural difference of the P-loop in the two apo forms may imply that the P-loop was quite flexible.

In the *Bs*IspD/CTP-Mg^2+^ complex, it was clearly seen that the P-loop had shifted away from the active site upon CTP binding (Supplementary Fig. S2), and it changed from beta-alpha (*β*-*α*) form to alpha-beta (*α*-*β*) form^32^. The C^α^ atoms of Gly11,Gln12 and Arg15 shifted 4.4 Å, 7.6 Å and 7.9 Å, respectively, in this way, the P-loop did not interact with residues Asp84, Asp104, Ala106, and Lys 209 anymore, the active pocket was open to accommodate CTP, the P-loop and the residues Lys209 and Ala106 were involved in the interactions with CTP (Fig. 4b). Moreover, conformational changes were also observed on L1 and L2 loops. The L1-loop rotated about 30° and moved about 2.4 Å, a cleft in 4 Å width was formed between the L1-loop and P-loop, favoring for the binding of the cytosine base of CTP. The concerted movement of L1-loop was also observed in the *Ms*IspD/CMP complex structure^33^. Meanwhile, it was observed the L2-loop shifted about 2 Å, the residue Thr211 was hydrogen bonded to the residue Arg15 in the P-loop (3.5 Å). This interaction between threonine and arginine was commonly seen in the structures of IspD/ligands complex, since the couple of residues were strictly conserved in the IspD enzymes. Therefore, concerted movement of the L2-loop may be also essential in the catalysis reaction.

In general, the flexibility of P-loop and adjacent loops suggested that they may function as a gate, regulating the binding of CTP and the dissociation of products. In addition, the residues Pro8-Gly13, Arg15, Lys209, Lys22, Asp81 and Asp104 were involved in the binding to CTP; these residues should be taken into account when the new inhibitors are designed.

### Conformational change of P-loop in the catalysis

The structural feature of IspD had been widely disscussed in the early studies, which producing excellent understanding on the reaction mechanism of IspD^34^. However, residues missing in P-loop of apo IspD hindered the intact description on the reaction cycle, here the P-loop was well defined in the structure of *Bs*IspD. It was right time to investigate the conformational change of the P-loop and the active site during the substrate binding and the process of the reaction.

**Figure 5:**
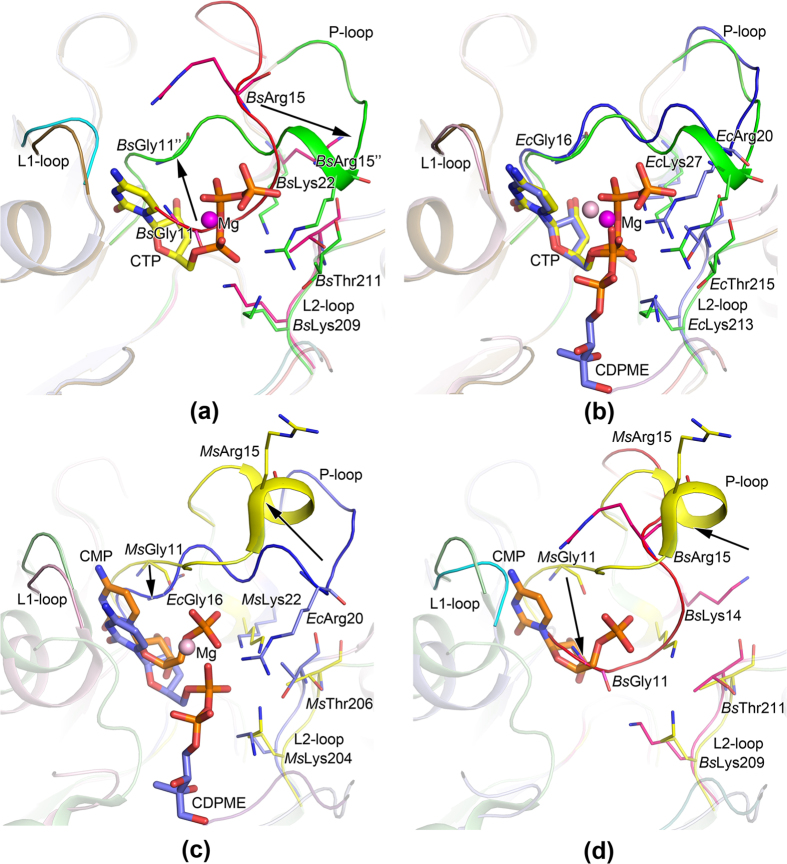
Superpositions of the IspD/substrate complex and *Bs*IspD. (**a**) *Bs*IspD/CTP-Mg^2+^ and *Bs*IspD; (**b**) *Bs*IspD/CTP-Mg^2+^ and *Ec*IspD/CDPME-Mg^2+^ (PDB entry 1ini), (**c**) *Ec*IspD/CDPME-Mg^2+^ and *Ms*IspD/CMP-Mg^2+^ (PDB entry 2xwm, chain B), (**d**) *Ms*IspD/CMP-Mg^2+^ and *Bs*IspD. The P-loops of *Bs*IspD, *Bs*IspD/CTP-Mg^2+^, *Ec*IspD/CDPME-Mg^2+^ and *Ms*IspD/CMP were colored in purple, green, blue and yellow, respectively. The conformational charge of the P-loop during the catalytic reaction was shown step by step with black arrow. The magnesium ion was show as sphere; CTP, CMP and CDPME and conserved residues in the binding pocket were shown in stick model.

The structures of IspD from various species are highly conserved; single subunit from these enzymes can be superimposed on *Bs*IspD with r.m.s.d in the range 1.6–2.2 Å for 199–220 pairs of C^α^ atoms (Supplementary Table S1). Based on structural similarity, typical structures of IspD and IspD/ligand complex have been compared in pairs, including the structures of *Bs*IspD, *Bs*IspD/CTP-Mg^2+^, *Ec*IspD/CDPME-Mg^2+^ and *Ms*IspD/CMP (Fig. 5).

As shown in Fig. 5a, the active site of *Bs*IspD was covered by P-loop; hydrogen bonding interactions were formed between P-loop and adjacent residues as described above. Upon CTP binding, conformational changes were observed on P-loop, L1-loop and L2-loop. The N-terminal half of the P-loop flipped upward from active pocket and the C-terminal half flipped down right, the active pocket was open to accommodate the CTP. A cleft was formed between L1-loop and P-loop, favoring for the binding of the cytosine base of CTP, at the same time, the residue Arg15 in the P-loop formed hydrogen bond to the residue Thr211 in L2-loop.

In *Ec*IspD/CDPME-Mg^2+^, the residues 13–16 of P-loop (residues 8–11 in *Bs*IspD), the residue Gly82 (Gly80 in *Bs*IspD) in L1-loop and the residue Ser88 (Ser86 in *Bs*IspD) interacted with the cytidine base and the ribose of CDPME in the same way as that in *Bs*IspD/CTP-Mg^2+^ (Fig. 5b). However, the difference between the α-phosphate groups in CDPME and CTP was observed, which implied that the α-phosphate group in CTP has shifted down to react with MEP. Due to the formation of products CDPME and PPi, the residues 17–19 in *Ec*IspD (residues 12–14 in *Bs*IspD) were too far to interact with the phosphate groups of CDPME. In addition, the P-loop region comprising residue 19–21 and the L2-loop in the *Ec*IspD/CDPME-Mg^2+^ have moved up about 1.2 Å. These results demostrated that concerted movements of the P-loop and L2-loop may be essential during the catalytic reaction.

So far, three structures of IspD/CMP complex had been reported^33^,^35^,^36^, namely *At*IspD/CMP, *Cj*IspDF/CMP (IspD and IspF fused protein coming from *Campylobacter jejuni*) and *Ms*IspD/CMP, the binding of CMP might mimic the intermediate product of the reaction catalyzed by IspD enzyme. Interestingly, in the chain A of *Ms*IspD/CMP complex, the CMP molecular shared the same interactions as the cytosine and α-phosphate of the CTP in *Bs*IspD/CTP-Mg^2+^. However, in the chain B of *Ms*IspD/CMP, conformational change in the P-loop was observed. The CMP shifted more than 2 Å as a rigid body^33^, the ribose did not interact with Ala101 and Ala102 anymore, and new hydrogen-bonding were formed between the phosphate group of CMP and the residues Ser12 & Gly13. As shown in Fig. 5c, the α-carbon atoms of the Arg15 and Gly11 in *Ms*IspD/CMP shifted about 7.6 Å and 2.4 Å away from that of the Arg20 and Gly16 in *Ec*IspD/CDPME, respectively, the P-loop lost interactions with L2-loop. Compared with that in *Ec*IspD/CDPME-Mg^2+^, the C-terminal half of the P-loop flipped up, the N-terminal half of the P-loop showed tendency shifting towards the active site. This may represent the intermediate state as the products was releasing from the active site (Fig. 5c).

In Fig. 5d, the α-carbon atoms of the Arg15 and Gly11 in *Bs*IspD shifted away from that in *Ms*IspD/CMP by ~4 Å and ~7.3 Å, respectively. Assuming *Ms*IspD/CMP as the intermediate state in the reaction, the further movement of the P-loop should lead to a fully close-up of the active pocket after the products releasing, then the IspD will be ready for next reaction cycle.

Based on the structures of *Bs*IspD in this study and previous structures of IspD/ligand complex^33^,^34^,^36^, a concerted movement model of the loops in the active pocket was proposed (Fig. 5). In the apo enzyme, the active site was in close state, upon the binding of the CTP, the P-loop flipped left-up and right-down to interact with the CTP, at the same time, the L1-loop slightly moved upward and the residue Arg15 in P-loop formed hydrogen bonding to adjacent Thr211 in L2-loop (in *Bs*IspD), this interaction would stabilize the open state of the active site during the catalysis reaction. Subsequently, the nucleophilic attack on the α-phosphate of the CTP by MEP would occur, the PPi and CDPME were produced. With the release of PPi and CDPME, the P-loop would shift toward to the active site, and the active site would be back in close status and ready for next turn.

In this model, the P-loop could move like a seesaw as described above, leading to the successive conformation change of the active site. The L1-loop and L2-loop can move in concert with the P-loop, the concerted movement of the loops in the active pocket would facilitate the opening of the pocket, the holding of substrates in place and releasing of the products.

### Enzyme activity

CTP hydrolysis activity of *Bs*IspD and *Ec*IspD was measured by using an inorganic pyrophosphatase-coupled assay. When CTP was the variable substrate, the *K*_*m*_ value was 124.8 *uM* and the *k*_*cat*_ value was 2180.9 min^−1^. And a *K*_*m*_ value of 132.8 *uM* and a *k*_*cat*_ value of 4274.4 min^−1^ were calculated when MEP was the variable substrate. Meanwhile, in the case of *Ec*IspD, the *K*_*m*_ value of 291.5 *uM* and a *k*_*cat*_ value of 2307.7 min^−1^ was calculated for CTP, and a *K*_*m*_ value of 230.9 *uM* and a *k*_*cat*_ value of 4770.2 min^−1^ for MEP (Table 2, Supplementary Fig. S4). The *K*_*m*_ and *k*_*cat*_ values of *Ec*IspD were basically similar to those published previously^37^. Some variations among these data may be due to the difference in experimental conditions and methods for data processing^37^,^38^,^39^.

In this study, the *Bs*IspD did exhibit a modest 1.5~2-fold higher catalytic efficiency than the *Ec*IspD, largely due to lower *K*_*m*_ values for both CTP and MEP, which implied higher substrate-binding affinity. Sequence alignment revealed that the *Bs*IspD shared 37% sequence identities to *Ec*IspD, the variation of the primary sequence may lead to the difference on the active pocket, such as the size and the shape, the hydrophobicity and the surface charge, which could affect the binding of the substrates. Structural analysis revealed the solvent accessible surface of CTP binding pocket in *Bs*IspD was 807 Å^2^ vs 882 Å^2^ in *Ec*IspD, the binding pocket in *Ec*IspD was more exposed to the solvent. In addition, It was observed that hydrogen bond was formed between the ε-amino group of Lys209 and the α-phosphate of CTP (Lys209 NZ-O1A = 2.8 Å) in this study, however, no such interaction was observed in *Ec*IspD (Lys213 of *Ec*IspD equivalent to Lys209 of *Bs*IspD). This interaction may also participate in stabilizing a pentacoordinate transition state or intermediate while the nucleophilic attack of the MEP phosphate on the α-phosphate of CTP occurred^37^.

The synthetic pathway for isoprene or other terpenoids were established in *E. coli*, but the yield should be further improved for commercial production. It has been reported that the activity of DXS and DXR from *B. subtilis* were higher than that in *E. coli*, because the genes of *dxs* and *dxr* from *B. subtilis* heterologously expressed in *E. coli* functioned more efficiently on the enhancement of isoprene production than the native ones^31^. Here the biochemical and structural data revealed that the *Bs*IspD was of a higher enzyme activity than that of *Ec*IspD. These results combined with the references^23^,^40^ may explain that *Bacillus* produced about 18-fold the level of isoprene than that produced by *E. coli*^41^.

## Conclusion

In conclusion, the crystal structures of *Bs*IspD and *Bs*IspD/CTP-Mg^2+^ had been determined in this study. It is the first time that the intact P-loop was visible in the apo structure of IspD enzymes, structural analysis revealed that the P-loop could play an important role in the reaction. It may flip like a seesaw to coordinate the processing of substrates binding, intermediate forming and products releasing. Moreover, two flexible loops may assist for the successive conformation change of the active pocket. Meanwhile, the IspD from *B. subtilis* could function more efficiently than *Ec*IspD. This study on the IspD enzyme will advance the understanding on the MEP pathway in *B. subtilis* and provide valuable information for the practice. Probably, the *B. subtilis* could be an ideal host cell for production of isoprenoids by synthetic biology, since the key enzymes of MEP pathway in the *B. subtilis* could function much better.

## Methods

### Expression and purification of IspD

The IspD gene of *Bacillus subtilis* 168 (DSM 23778, DSMZ, Germany) was amplified by PCR from genomic DNA with the 5′/3′ specific primers which introduced *Bam*HI site and *Xho*I site, respectively. The 699 bp amplification products were digested and cloned into the vector of pGEX-6P-1, the gene sequence was confirmed by DNA sequencing. The recombinant plasmid was designated as pGEX-6P-1-*Bs*IspD. The recombinant plasmid was transformed into competent *E. coli* DH5α. Bacteria cells were grown in LB broth supplemented with 100 μg/ml ampicillin. The culture was incubated at 37 °C with vigorous shaking. At an optical density (600 nm) of 0.5~0.6, IPTG was added to a final concentration of 0.3 mM, and the culture was further incubated at 16 °C for 14~16 h. The cells were harvested by centrifugating, the cell pellets were re-suspended in lysis buffer (25 mM Tris-HCl pH 8.0, 50 mM NaCl, 1 mM DTT) and sonicated on ice. The crude fluid was centrifuged at 11,800 × g at 4 °C for 40 min to remove cellular debris. The IspD was purified from the supernatant by GST Glutathione Sepharose^TM^ 4 Fast Flow column (GE Healthcare), and the GST tag was removed by Prescission Protease (PPase) at 4 degree overnight. The eluted IspD enzyme was further purified by the combination of the Resource Q (GE Healthcare) anion-exchange column and Superdex 75 (GE Healthcare) size-exclusion column. The protein fractions were pooled and determined to be ~99% pure by SDS-PAGE. The protein concentration was determined by Bradford method using bovine serum albumin (BSA) as standard. The purified protein was aliquoted for storage at −80 °C.

Meanwhile, the *E. coli* IspD gene encoding CDPME synthetase was PCR amplified from *E. coli K12* and inserted into the pET22b expression vector. C-terminal His_6_-tagged protein was expressed in *E. coli* BL21 (DE3) cells. Tagged CDPME synthetase was purified using a Ni^2+^-NTA (GE Healthcare) column. The histidine tag was removed by thrombin enzyme. Therefore the no-tagged protein was purified to greater than 99% homogeneity by anion exchange column and gel filtration column.

### Enzyme assay

The activity of IspD in the conversion of MEP and CTP to CDPME and PPi was evaluated in a coupled assay^39^. When the product PPi is hydrolyzed by inorganic pyrophosphatase, the Pi forms a complex with malachite green that can be detected at 670 nm^42^. For each determination a standard curve was made at the concentration gradient of potassium dihydrogen phosphate standards (0~50 μM). To carry out the colorimetric assay, one volume of dye reagent was mixed with four volumes of sample and incubated at 30 °C for 10 min. Absorbances at 670 nm were recorded, corrected for blank, and plotted.

The standard IspD activity reaction mixture contained 0.1 M Tris-HCl (pH 8.0), 1 mM MgCl_2_, 0.125 mM MEP, 0.2 mM CTP, 1 mM DTT, 100 mU/ml of inorganic pyrophosphatase, and recombinant IspD in a final volume of 200 μl. The control reaction contained all the components except IspD. Reactions were carried out at 30 °C and started by the addition of the IspD. After the reaction took place, 40 μl of reaction mixture was taken and mixed with 120 μl of distilled water and 40 μl of dye reagent. Plates were incubated at 30 °C under stirring and the absorbance at 670 nm was measured in a Thermo Dynatech microtiter plate reader. Each measurement was repeated at least three times.

The apparent *K*_*m*_ values of IspD were determined with varying MEP concentrations from 10 to 400 μM and a fixed amount of IspD (0.1 μg). The effect of the CTP concentration on the reaction rate catalyzed by IspD was studied by using different CTP concentrations ranging from 10 to 500 μM and 0.1 μg of IspD. Kinetic constants were obtained by fitting the experimental data to the appropriate rate equations by nonlinear regression^42^.

### Protein Crystallization and Data Collection

Crystallization trials were performed using the hanging-drop vapour-diffusion method at 18 °C. Hampton Crystallization Kits were used to get the appropriate crystallization conditions. Crystals of the *Bs*IspD proteins (at 8 mg/ml in 25 mM Tris-HCl pH 8.0, 0.1 M NaCl) were obtained from two different crystallization conditions.

Condition I was obtained from the solution of 0.2 M trimethylamine N-oxide dehydrate, 0.1 M Tris (pH 8.5), 20% (v/v) polyethylene glycol monomethyl ether 2,000. Condition II was obtained from the solution of 0.2 M magnesium chloride, 0.1 M HEPES (pH 7.5), 25% (v/v) Polyethylene glycol 3,350. The crystallization trials were optimized by adding the *β*-OG and combination with the seeding method. In addition, *Bs*IspD proteins were incubated with 10 mM MgCl_2_ and 10 mM CTP for 1 h on ice before trays setup, the crystals of *Bs*IspD/CTP-Mg^2+^ complex were obtained in the condition II.

The hanging drops containing crystals were dehydrated to the reservoir solution plus 15% of glycerol overnight. Then the crystals were picked from the solution using nylon loops and frozen in liquid nitrogen. X-ray diffraction data of *Bs*IspD crystals was collected at 100 K using synchrotron radiation at Beijing Synchrotron Radiation Facilities (BSRF), Shanghai Synchrotron Radiation Facilities (SSRF) and National Center for Protein Science Shanghai (NCPSS). Data sets were processed and scaled by HKL2000^43^. Details are presented in Table 1.

### Structure determination and refinement

The structures of *Bs*IspD were elucidated by molecular replacement using PHASER^44^ from the CCP4 program suite^45^. The starting model was the monomer of IspD enzyme from *A.Thaliana* (*At*IspD) (PDB entry 2yc3), which shared 38% sequence identity with the *Bs*IspD and served as a good model for the structure solution of the two crystal forms. Only one solution was evident. Refinement was performed using the maximum likelihood functions implemented in REFMAC5^46^, while model building and improvement were achieved with COOT^47^. Solvent molecules were positioned after a few cycles of refinement. Isotropic refinement of the atomic displacement parameters was performed for all atoms. The stereochemistry was checked with the program PROCHECK^48^. Details of the overall refinement and final quality of the models were shown in Table 1. The program PyMOL (http://www.pymol.sourceforge.net/) was used to prepare structural figures.

### Solvent accessible surface calculation

The solvent accessible surface (Å^2^) of CTP binding pocket was analyzed by using CASTP server^49^.

## Additional Information

**Accession codes:** The atomic coordinates and structure factors have been deposited in the Protein Data Bank with accession codes 5ddt (Apo form I), 5ddv (Apo form II) and 5hs2 (IspD/CTP-Mg^2+^ complex), respectively.

**How to cite this article:** Jin, Y. *et al*. A structural and functional study on the 2-C-methyl-D-erythritol-4-phosphate cytidyltransferase (IspD) from *Bacillus subtilis*. *Sci. Rep.* **6**, 36379; doi: 10.1038/srep36379 (2016).

**Publisher’s note:** Springer Nature remains neutral with regard to jurisdictional claims in published maps and institutional affiliations.

## Supplementary Material

Supplementary Information

## Figures and Tables

**Figure 1:**
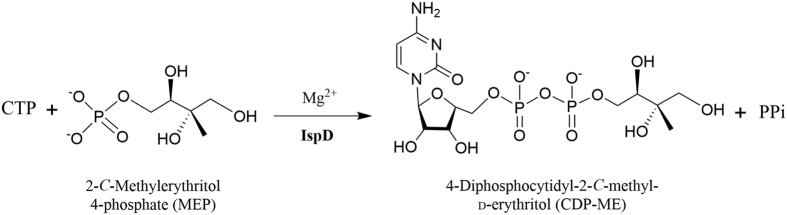
The third step in the MEP pathway is catalyzed by IspD.

**Table 1:** Data collection and refinement statistics.

Crystallization Condition	Apo form I	Apo form II	IspD/CTP-Mg^2+^ complex
	I	II	II and co-crystallization with 10 mM CTP and 10 mM MgCl_2_
Data collection			
Synchrotron beamline	SSRF BL17U1	BSRF 3W1A	NCPSS BL18U1
Wavelength(Å)	0.97915	1.0000	0.9778
Space group	*P*2_1_2_1_2_1_	*P*2_1_2_1_2	*P*2_1_
Unit-cell parameters			
a, b, c (Å,)	63.34,77.95,91.47	64.47,85.73,45.82	64.45,49.43,75.37
α,β,γ(°)	90,90,90	90,90,90	90,101.4,90
Monomers per asymemetric unit	2	1	2
Resolution	30.0 − 1.80 (1.86 − 1.80)^a^	28.2 − 2.30 (2.38 − 2.30)^a^	29.6 − 1.90 (1.93 − 1.90)^a^
No. of unique reflections	42387 (4225)^a^	11687 (1165)^a^	37021 (1811)^a^
Redundancy	3.6 (3.6)^a^	4.5 (4.3)^a^	2.8 (2.8)^a^
Completeness (%)	99.0 (100)^a^	98.6 (99.4)^a^	98.8 (99.0)^a^
Mean I/σ	21.7 (3.7)^a^	23.0 (3.9)^a^	6.6 (2.3)^a^
R_merge_(%)	5.2 (34.9)^a^	5.7 (31.6)^a^	14.1 (53.5)^a^
Refinement			
Reflections (working/test)	40202/2142	11071/558	35210/1798
R_work_/R_free_(%)	18.0/21.6	20.8/25.7	20.4/25.2
Number of residues			
Protein(A/B)	232/232	223 (13–14,226–232 miss)	226 (227–232 miss)/ 215(10–20,227–232 miss)
CTP/Mg	0/0	0/0	1/1
Waters	458	68	208
Average B factor (Å^2^)			
Main Chain/Side Chain	A24.4/30.3, B24.9/30.9	42.7/47.8	A33.4/40.1,B53.7/59.5
CTP/MG	—	—	24.6/24.7
Waters	37.0	42.8	43.4
Ramachandran plot(%)			
Favoured	98.5	98.6	98.6
Allowed	1.5	1.4	1.4
R.m.s deviations			
Bond lengths(Å)	0.015	0.015	0.014
Bond angles(°)	1.672	1.702	1.606

^a^The values in parenthesis means those for the highest resolution shell.

**Table 2:** Kinetic parameters for *Bs*IspD and *Ec*IspD.

	MEP			CTP			References
	*K*_*m*_ (μM)	*k*_*cat*_ (min^−1^)	*k*_*cat*_/*K*_*m*_ (mM^−1^min^−1^)	*K*_*m*_ (μM)	*k*_*cat*_ (min^−1^)	*k*_*cat*_/*K*_*m*_ (mM^−1^min^−1^)	
*Bs*IspD	124.8 ± 19	2180.9	17475.0	132.8 ± 29	4274.4	32186.7	This study
*Ec*IspD	291.5 ± 44	2307.2	7915.1	230.9 ± 69	4770.2	20660.4	This study
*Ec*IspD	370 ± 60	2904 ± 660	7849	760 ± 60	3246 ± 1680	4271	Richard *et al*.^37^
*Ec*IspD	32 ± 3	1008 ± 12	31500	ND	ND	ND	Cane *et al*.^38^
*Ec*IspD	61 ± 14	ND	ND	58 ± 6	ND	ND	Bernal *et al*.^39^

ND is defined as not determined.
