# Supplementary material for: A structural and functional study on the 2-C-methyl-d-erythritol-4-phosphate cytidyltransferase (IspD) from Bacillus subtilis
Source: Sci Rep. 2016 Nov 8;6:36379. doi: 10.1038/srep36379 (PMC5099578; doi:10.1038/srep36379)
Supplement: Supplementary Information [file srep36379-s1.doc]

**Supplementary Materials**

**A structural and functional study on the 2-C-methyl-d-erythritol-4-phosphate cytidyltransferase (IspD) from *Bacillus subtilis***

Yun Jin^1，2，4^, Zhongchuan Liu ^1，2^, Yanjie Li^1，2，^Weifeng Liu^3^, Yong Tao^3^, Ganggang Wang ^1，2*^

*^1^Key Laboratory of Environmental and Applied Microbiology, Chengdu Institute of Biology, Chinese Academy of Sciences, Chengdu, 610041, China;*

*^2^Key Laboratory of Environmental Microbiology of Sichuan Province, Chengdu, 610041, China;*

*^3^Chinese Academy of Sciences Key Laboratory of Microbial Physiological and Metabolic Engineering, Institute of Microbiology, Chinese Academy of Sciences, Beijing 100101, People's Republic of China;*

*^4^University of Chinese Academy of Sciences, Beijing, 100049, China.*

*Corresponding author’s:

SEND CORRESPONDENCE TO*:*

Ganggang Wang

Key Laboratory of Environmental and Applied Microbiology, Chengdu Institute of Biology, Chinese Academy of Sciences, Chengdu, 610041, China

Tel: 86-28-82890828; E-mail: wanggg@cib.ac.cn


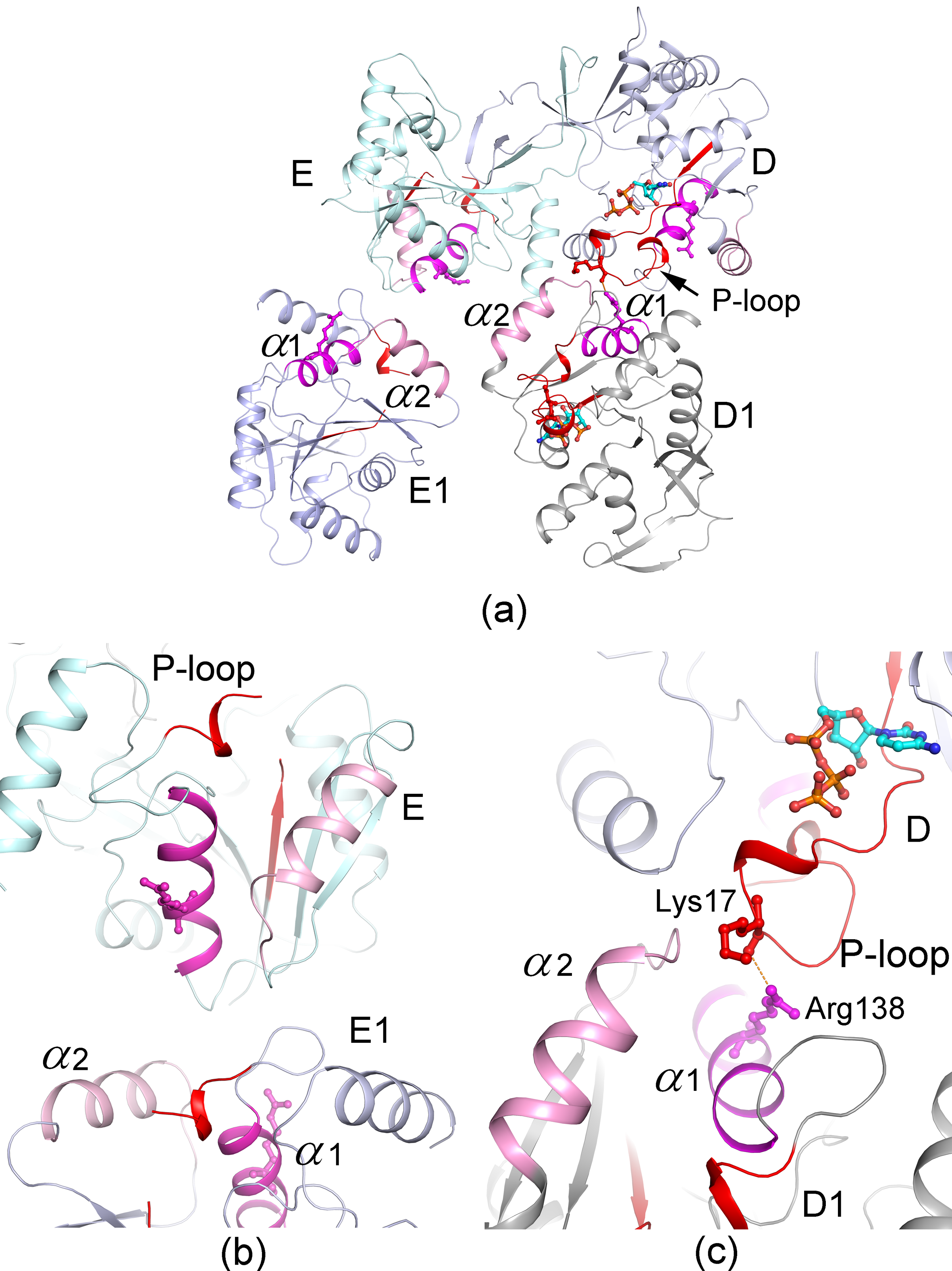


**Figure S1**

**Molecule packing in the structure of *Bs*IspD/CTP-Mg^2+^ complex.** (a) Adjacent molecules (D1, E1) were packed with D/E dimmer. (b) Molecule packing between molecules E and E1 (ligand free). (c) Molecule packing between molecules D and D1 (CTP binding). The CTP molecule was shown in stick. The α1 and α2 helices in each subunit were colored in magenta and pink, P-loop region was colored in red.


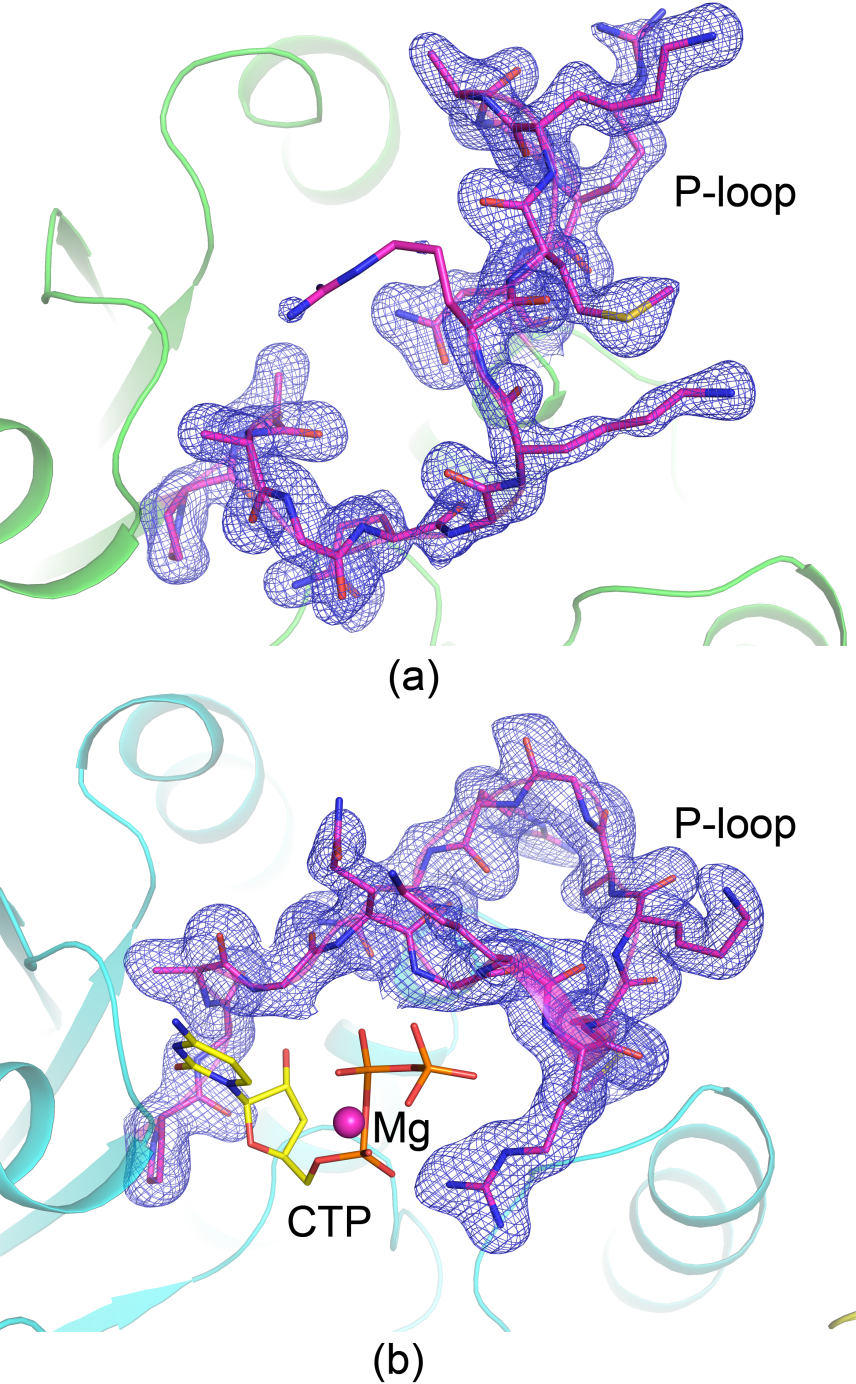


**Figure S2**

**2Fo-Fc electron density map of the P-loop in the structures of (a) Apo form I and (b) *Bs*IspD/CTP-Mg^2+^ complex.** The 2Fo-Fc electron density map (1.0σ level) was represented in blue. The P-loop and the CTP molecule were shown in stick mode, and magnesium ion was shown in sphere.


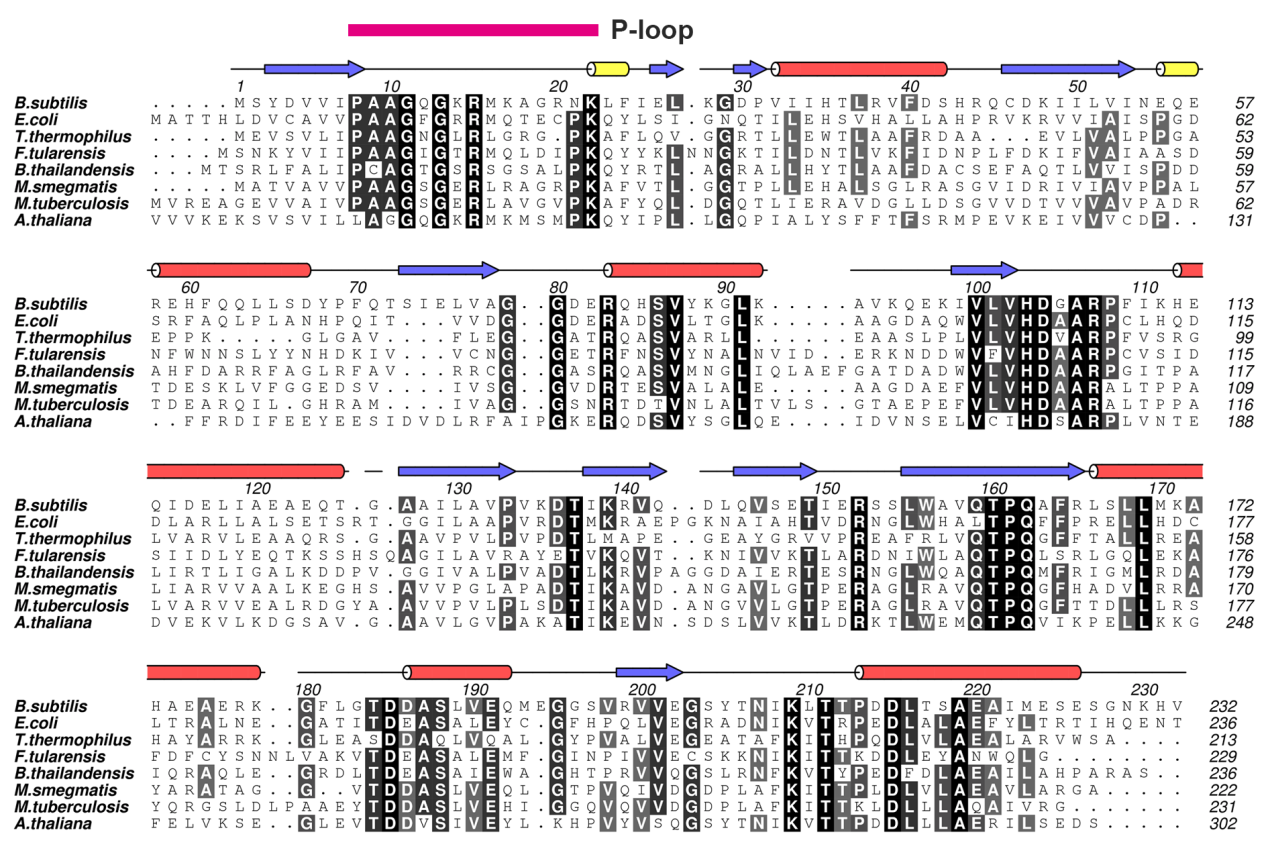


**Figure S3**

**Sequence alignment.** Sequences of IspD of *B.subtillis*, *E.coli*, *Thermus thermophilus HB8*, *Francisella tularensis*, *Burkholderia thailandensis E264*, *Mycobacterium smegmatis*, *M.tuberculosis* and *A.thaliana* (UniProt identifiers Q06755, Q46893, Q72GN3, Q2A280, Q2SWT6, A0R560, C6DMN4, P69834, respectively) were aligned with *MUSCLE* ^1^ and edited by hand to match the structural similarity where appropriate by using *ALINE* ^2^. Identical and similar residues were highlighted in black and grey, respectivey. *α*-Helix and *β*-Strand are marked by red pillar and blue arrow, respectively. The P-loop area was marked by peach rectangle.


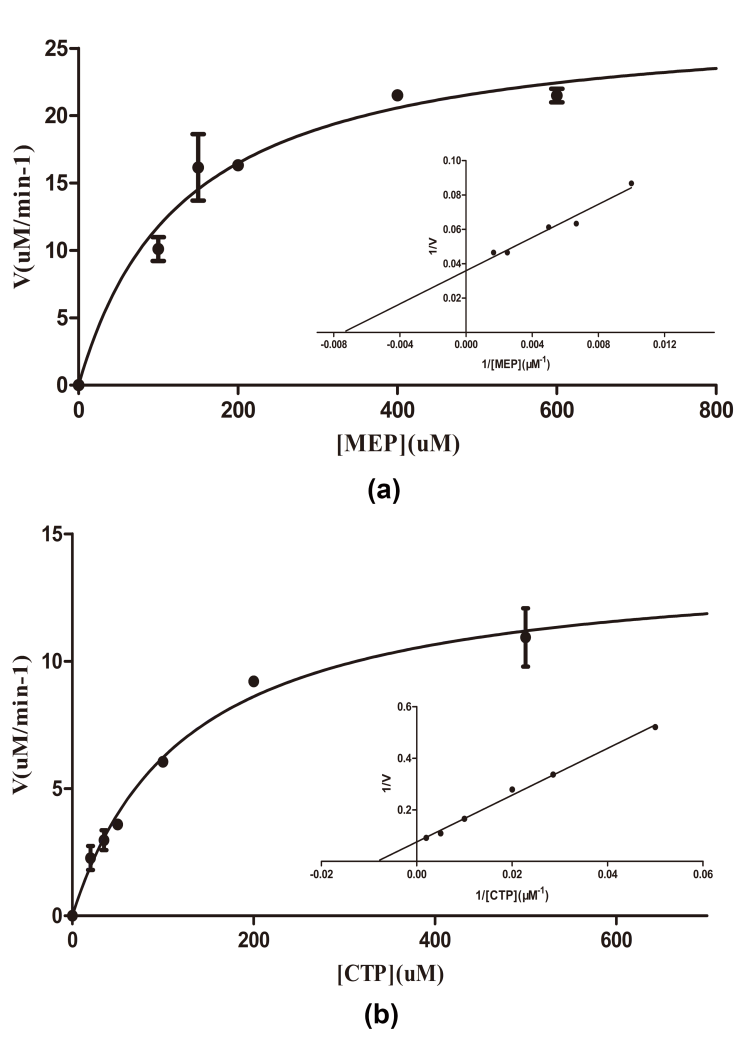


**Figure S4** **Enzyme kinetic parameters of *Bs*IspD.** (a) Kinetic analysis of *Bs*IspD activities toward MEP. Initial velocities of *Bs*IspD were measured in the presence of varying concentrations of MEP. CTP (0.2 mM) was present for all measurements. (b) Kinetic analysis of *Bs*IspD activities toward CTP. Initial velocities of *Bs*IspD were measured in the presence of varying concentrations of CTP. The concentration of MEP was constant at 0.125 mM. The error bars represented the standard deviation of three replicates. The inset showed Lineweaver–Burk plot for the determination of Michaelis kinetic constants (Vmax, *Km*).

**Table S1**

**Structural comparisons with the chain A of *Bs*IspD and *Bs*IspD/CTP-Mg^2+^ complex**

| Enzyme | Z | R.m.s.d.  (Å) | Lali | nres | %id | Ligands | Resolusion  (Å) | Missing location in P-loop | PDB number  and reference |
| --- | --- | --- | --- | --- | --- | --- | --- | --- | --- |
| **Apo form** |  |  |  |  |  |  |  |  |  |
| *B.subtillis* IspD | 42.2 | 0.0 | 232 | 232 | 100 | apo | 1.80 | 0 | 5ddt * |
| *B.subtillis* IspD | 36.2 | 0.8 | 223 | 223 | 100 | apo | 2.30 | 13-14 | 5ddv * |
| *E.coli* IspD | 26.1 | 1.9 | 203 | 207 | 35 | apo | 1.80 | 16-25 | 1vgt ^3^ |
| *E.coli* IspD | 27.3 | 1.7 | 208 | 213 | 35 | apo | 1.60 | 16-26 | 1inj ^4^ |
| *E.coli* IspD | 27.5 | 1.8 | 211 | 215 | 35 | apo | 2.40 | 16-24 | 1h3m ^5^ |
| *E.coli* IspD | 26.5 | 1.9 | 210 | 214 | 34 | apo | 2.80 | 15-24 | 1vgu ^3^ |
| *Neisseria.gonorrhoeae* IspD | 25.0 | 1.9 | 203 | 212 | 34 | apo | 2.40 | 13-22 | 1vgw ^3^ |
| *A.thaliana* IspD | 28.1 | 1.9 | 209 | 214 | 36 | apo | 1.50 | 87-96 | 4nai ^6^ |
| **IspD/ligand complex** |  |  |  |  |  |  |  |  |  |
| *B.subtillis* IspD | 40.9 | 0.1 | 226 | 226 | 100 | CTP,Mg2^+^ | 1.9 | 0 | 5hs2 * |
| *E.coli* IspD | 30.6 | 1.8 | 220 | 225 | 35 | CTP,Mg^2+^ | 1.50 | 0 | 1i52 ^4^ |
| *E.coli* IspD | 30.6 | 1.6 | 219 | 225 | 35 | CDPME, Mg^2+^ | 1.80 | 0 | 1ini ^4^ |
| *E.coli* IspD | 27.9 | 1.7 | 211 | 220 | 35 | 1,2-Propanediol | 1.90 | 16-33 | 3n9w ^7^ |
| *M. smegmatis* IspD | 28.7 | 2.1 | 216 | 220 | 37 | CMP | 1.80 | 0 | 2xwm ^8^ |
| *M. smegmatis* IspD | 29.4 | 1.9 | 216 | 221 | 37 | CTP,Mg^2+^ | 1.49 | 0 | 2xwl ^8^ |
| *M. tuberculosis* IspD | 28.9 | 1.8 | 214 | 221 | 34 | CTP,Mg^2+^ | 2.90 | 0 | 2xwn ^8^ |
| *C. jejuni* IspDF | 25.5 | 2.0 | 204 | 369 | 27 | CMP | 2.30 | 0 | 1w55 ^9^ |
| *A.thaliana* IspD | 26.4 | 2.2 | 204 | 212 | 35 | CMP | 2.00 | 88-94 | 1w77 ^10^ |

* is represented for this study. Z score is the degree of structural similarity in standard deviations above that expected as reported by the *DALI* server ^11,12^. R.m.s.d. is the positional root-mean-square deviation of superimposed C atoms. Lali is the total number of equivalent residues. nres is the length of the entire chain of the equivalent structure. %id is the percentage sequence identity over equivalent positions.

**Reference**

1 Edgar, R. C. MUSCLE: multiple sequence alignment with high accuracy and high throughput. *Nucleic. Acids. Res.* **32**, 1792-1797 (2004).

2 Bond, C. S. & Schüttelkopf, A. W. ALINE : a WYSIWYG protein-sequence alignment editor for publication-quality alignments. *Acta. Crystallogr. D.* **65**, 510–512 (2009).

3 Badger, J. *et al.* Structural analysis of a set of proteins resulting from a bacterial genomics project. *Proteins.* **60**, 787-796 (2005).

4 Richard, S. B. *et al.* Structure of 4-diphosphocytidyl-2-C-methylerythritol synthetase involved in mevalonate-independent isoprenoid biosynthesis. *Nat. Struct. Mol. Biol.* **8**, 641-648 (2001).

5 Kemp, L. E., Bond, C. S. & Hunter, W. N. Structure of a tetragonal crystal form of *Escherichia coli* 2-C-methyl-D-erythritol 4-phosphate cytidylyltransferase. *Acta. Crystallogr. D.* **59**, 607-610 (2003).

6 Kunfermann, A. *et al.* Pseudilins: Halogenated, Allosteric Inhibitors of the Non-Mevalonate Pathway Enzyme IspD. *Angew. Chem. Int. Edit.* **53**, 2235-2239 (2014).

7 Behnen, J. *et al.* Experimental and Computational Active Site Mapping as a Starting Point to Fragment-Based Lead Discovery. *ChemMedChem.* **7**, 248-261 (2012).

8 Björkelid, C. *et al.* Structural and functional studies of mycobacterial IspD enzymes. *Acta. Crystallogr. D.* **67**, 403-414 (2011).

9 Gabrielsen, M. *et al.* Biosynthesis of isoprenoids: a bifunctional IspDF enzyme from *Campylobacter jejuni*. *Eur. J. Biochem.* **271**, 3028-3035 (2004).

10 Gabrielsen, M. *et al.* The crystal structure of a plant 2C-methyl-D-erythritol 4-phosphate cytidylyltransferase exhibits a distinct quaternary structure compared to bacterial homologues and a possible role in feedback regulation for cytidine monophosphate. *FEBS. J.* **273**, 1065-1073 (2006).

11 Holm, L. & Park, J. DaliLite workbench for protein structure comparison. *Bioinformatics* **16**, 566-567 (2000).

12 Hasegawa, H. & Holm, L. Advances and pitfalls of protein structural alignment. *Curr. Opin. Struc. Biol.* **19**, 341-348 (2009).
